# What are the macrophages and stellate cells doing in pancreatic adenocarcinoma?

**DOI:** 10.3389/fphys.2015.00125

**Published:** 2015-05-15

**Authors:** Stephen J. Pandol, Mouad Edderkaoui

**Affiliations:** ^1^Departments of Medicine and Biological Sciences, Cedars-Sinai Medical CenterLos Angeles, CA, USA; ^2^Veterans Affairs Greater Los Angeles Healthcare System and University of California, Los AngelesLos Angeles, CA, USA

**Keywords:** stellate cells, macrophages, pancreatic cancer, periostin, TGF-beta

## Abstract

Pancreatic ductal adenocarcinoma is a devastating disease characterized by a dense desmoplastic stroma. Chemo- and radio-therapeutic strategies based on targeting cancer cells have failed in improving the outcome of this cancer suggesting important roles for stroma in therapy resistance. Cells in the tumor stroma have been shown to regulate proliferation, resistance to apoptosis and treatments, epithelial to mesenchymal transition (EMT) and stemness of cancer cells. Stellate cells in their activated state have been thought over the past decade to only have tumor promoting roles. However, recent findings suggest that stellate cells may have protective roles as well. The present review highlights the latest findings on the role of two major components of tumor stroma, pancreatic stellate cells and macrophages, in promoting or inhibiting pancreatic cancer, focused on their effects on EMT and cancer stemness.

## Introduction

Pancreatic ductal adenocarcinoma (PDAC) is the fourth leading cause of cancer-related death in the United States. The 5-year survival rate remains at 5% making it one of the worst outcomes among cancers (Siegel et al., 2012).

PDAC is unique among solid tumors because of the extremely dense desmoplastic stroma that surrounds the cancer cell glands of this tumor. In fact, the majority of the tumor volume is made of stroma compared to a much smaller volume occupied by the cancer cells. Understanding of the populations of the cells present in the stroma is needed to better target the correct sub-populations.

## Stellate cells in the context of pancreatic cancer

Pancreatic stellate cells (PSCs) were first isolated in 1998 by two groups- one from Australia and one Germany (Apte et al., 1998; Bachem et al., 1998). Since then extensive work has been done to understand the role of PSC in promoting pancreatic fibrosis and cancer. There has been general agreement for over a decade that the PSCs promote pancreatic cancer growth, metastasis and resistance to treatment, with observed association between strong stroma and worse outcome (Erkan et al., 2008; Beatty et al., 2011). Indeed, stellate cells promote the growth of cancer cells in sub-cutaneous mouse models of pancreatic cancer (Bachem et al., 2005). One mechanism through which PSCs mediate their pro-cancer effect is through secretion of large amounts of extracellular matrix proteins, responsible in large part for the fibrotic reaction characterizing human PDAC (Apte et al., 2004) as well as for inducing resistance to apoptosis in the cancer cells (Edderkaoui et al., 2005). Activated PSCs secrete growth factors, metallo-proteases, and cytokines shown to stimulate cancer cell proliferation, migration and metastasis of the pancreatic cancer cells (Wehr et al., 2011).

## Stellate cells regulate epithelial-mesenchymal transition (EMT) and stemness of the cancer cells

A major cause of the poor outcome for patients with pancreatic cancer is the ability of the cancer cells to metastasize and to develop resistance to treatments mainly through development of cancer stemness characteristics. EMT is a process by which epithelial cells lose their cell polarity and cell-cell adhesion while gaining migratory and invasive properties to become mesenchymal stem cells. The importance of the process of EMT is that it is responsible for both metastasis and therapy resistance through development of cancer stemness (Mani et al., 2008; Arumugam et al., 2009). Cancer stem cells markers such as ALDH1A1, ABCG2, and nestin are highly expressed in metastatic cancer cells compared to non-metastatic cancer cells in animal models of pancreatic cancer. The metastatic cells show strong expression of EMT markers indicating the association between EMT, cancer stemness and metastasis (Matsuda et al., 2014).

Recently, the role of PSCs in up-regulating EMT has become more evident. Co-culture of cancer cells with PSCs induces a fibroblast-like appearance of the cancer cells (Kikuta et al., 2010). The cancer cells assume fibroblast characteristics such as increased migration; and expression of mesenchymal markers Vimentin, Snail-1 and Zeb; and decrease expression of epithelial markers in the cancer cells (Kikuta et al., 2010; Mizuuchi et al., 2014). The mechanisms underlying the promotion of EMT are still under investigation and are not established. Because PSCs produce significant amounts of Transforming growth factor-beta (TGF-β) (Shek et al., 2002), there has been interest in the role of TGF-β in EMT. In one study, the PSC-induced EMT induction was not altered by treatment with anti-TGF-β-neutralizing antibody, suggesting no role of TGF-β in this process (Kikuta et al., 2010). Differently, another study showed that specific neutralizing anti-TGF-β prevented EMT, cancer stem cells phenotype, and tumorigenicity (Al-Assar et al., 2014).

There is accumulating evidence that small non-coding microRNAs (miRNAs) mediate the interaction between PSCs and cancer cells. Co-culture of pancreatic cancer cells with PSCs led to increased expression of miR-210. PSCs-induced miR-210 up-regulation was inhibited by inhibitors of ERK and PI3K/Akt pathways. Inhibition of miR-210 expression decreased the expression of Vimentin and Snail-1 as well as cell migration, and increased the membrane-associated expression of β-catenin in Panc-1 cells co-cultured with PSCs indicating its role in regulating EMT and migration of the pancreatic cancer cells (Takikawa et al., 2013).

The role of hypoxia, which is highly present in solid tumors, on tumor promotion was highly investigated in the last few years, Hypoxia increases the activity of PSCs *in vitro* leading to its migration, secretion of collagen I and VEGF (Masamune et al., 2008; Erkan et al., 2009). The fibrosis-related gene connective tissue growth factor (CTGF/CCN2) protects cells from hypoxia-mediated apoptosis, providing an *in vivo* selection for tumor cells that express high levels of CTGF/CCN2. Indeed, CTGF/CCN2 expression and secretion was increased in hypoxic pancreatic tumor cells *in vitro*, and co-localized with hypoxia in pancreatic tumor xenografts and clinical pancreatic adenocarcinomas (Bennewith et al., 2009).

PSCs enhance the cancer stem cell phenotype of pancreatic cancer cells by inducing the expression of genes such as ABCG2, Nestin and LIN28 leading to an enhanced radio-resistance of pancreatic cancer cells in *in vitro* and *in vivo* systems of pancreatic cancer (Hamada et al., 2012; Al-Assar et al., 2014). The expression of several EMT and cancer stem cell markers is associated with significant *in vivo* tumorigenicity.

Very importantly, PSCs show greater activity when isolated from patients after undergoing chemo-radiation therapy as measured by the ability of PSCs to migrate, expand and contract (Cabrera et al., 2014). This data together with the high level of resistance to treatments developed in pancreatic cancer patients suggest that PSC activity may contribute to the development of resistance to therapy in the cancer cells. Indeed, PSCs were found to promote *in vitro* sphere formation and invasiveness of pancreatic cancer stem cells in an activin/ Alk4 receptor -dependent manner (Lonardo et al., 2012). They also stimulated metastasis and the ability to form colonies in mouse orthotopic model of pancreatic cancer (Hwang et al., 2008). In another study, PSCs mediated radioprotection of pancreatic cancer cells in a β1 integrin dependent manner (Mantoni et al., 2011).

Clinical treatment of pancreatic cancer patients with Nab-paclitaxel combined with gemcitabine significantly decreased tumor growth and collagen staining as well as decreased the level of activated stellate cells (Alvarez et al., 2013). There was no association between the level of the secreted protein acidic and rich in cysteine (SPARC) and metastasis or the effect of Nab-paclitaxel (Schneeweiss et al., 2014).

## Controversial role of stellate cells

More recently, emerging data is challenging the established PSC pro-cancer role and provides strong evidence for an anti-cancer role of PSCs.

Ozdemir et al. (2014) showed that deletion of activated stellate cells in transgenic mice led to invasive and undifferentiated tumors, enhanced EMT, cancer stemness and decreased survival. The deletion of stellate cells resulted in decreases in both non-invasive precursor pancreatic intraepithelial neoplasia (PanIN) and the PDAC stage of the disease. Importantly, the authors observed an association between fewer activated stellate cells in the tumors with reduced survival in PDAC patients confirming their animal observations (Ozdemir et al., 2014). Finally, depletion of PSCs induced suppression of immune surveillance with increased CD4^+^Foxp3^+^Tregs in mouse tumors (Ozdemir et al., 2014). Of note, CD4^+^Foxp3^+^Tregs are cells of the immune system that suppress immune responses allowing the cancer cells to survive the immune checkpoint.

Simultaneously with this study, another study confirmed this data showing that depletion of stromal cells from pancreatic tumors, through genetic or pharmacological targeting of the Sonic hedgehog (SH) pathway, results in a reduced stromal content as expected, but led to a poorly differentiated histology, increased vascularity and proliferation, and reduced survival in an animal model of pancreatic cancer. Of note, SH drives the formation of a fibroblast-rich desmoplastic stroma in the tumor (Rhim et al., 2014).

Very importantly, the secretory protein periostin that has been suggested to function as a cell adhesion molecule and promote the invasiveness or growth rate of tumors is highly expressed by PSCs compared to cancer cells in humans (Erkan et al., 2007; Kanno et al., 2008). In addition, Recombinant periostin increased activation of the PSCs. These effects were reversed by silencing periostin expression and secretion by small interfering RNA transfection. Periostin stimulated cancer cell growth and induced their chemoresistance as well as resistance to starvation and hypoxia (Erkan et al., 2007).

This data suggest that PSC activation is a response by the body to prevent cancer development. The mechanism mediating this effect is not well known.

However, data from Kanno et al. (2008) showed that high concentration of recombinant periostin promoted cell migration of the cancer cells mediated by Akt kinase activation. They also found that lower doses of periostin had opposite effect causing expression of epithelial phenotype markers and reduction in mesenchymal markers resulting in reduced cell migration. The findings suggest that periostin has concentration-dependent dual effects on the development of pancreatic cancer (Kanno et al., 2008), especially on EMT and migration of the cancer cells. In the Erkan et al. study, periostin induced activation of the PSCs but decreased their invasiveness (Erkan et al., 2007). Periostin is highly expressed by PSC and its level may be a key regulator of the pro-EMT/cancer and the anti-EMT/cancer effects of PSC. That is, high expression of periostin by PSC will induce EMT, migration metastasis and stemness of the cancer cells; whereas, low doses of periostin will do the opposite.

The findings listed above indicate that there are potentially divergent roles for stellate cells in pancreatic cancer. That is, there may be pro-cancer, neutral, and anti-cancer effects of this stromal component. One possibility is that there are sub-populations and/or sub-phenotypes of PSCs that play different roles in the promotion or prevention of PDAC. The level of periostin expressed by PSC could be a good marker to start with. The different sub-phenotypes of PSCs may be affected by the other tumor microenvironment components. Targeting all of the PSCs together may not necessarily lead to an anti-cancer effect. Further studies are needed to better understand the potential divergent roles of PSCs before defining a treatment strategy based on targeting PSCs.

## Role of macrophages in promoting pancreatic cancer

In addition to the PSCs, macrophages are a major component of the pancreatic tumor microenvironment. The link between inflammation and pancreatic cancer pathogenesis is well established (Yadav and Lowenfels, 2013).

Recently published studies suggest that the macrophages infiltrating the pancreas drive the acinar to ductal metaplasia, a key early process in pancreas carcinogenesis. Pancreatic acinar cells have the capacity to undergo metaplasia to a ductal cell phenotype in the setting of acute or chronic inflammation, representing an important link to PDAC. The process involves inflammatory cytokines such as RANTES and tumor necrosis factor-alpha (TNF-α) and inflammatory intracellular signals such as nuclear factor-κB (NF-κB) as well as growth factors such as TGF-α and epidermal growth factor-receptor (EGFR) (De Lisle and Logsdon, 1990; Sandgren et al., 1990; Song et al., 1999; Means et al., 2003). Genetically engineered mouse models using mutant Kras support the concept that acinar to ductal metaplasia precedes PanIN and PDAC development (Song et al., 1999; Means et al., 2005). Importantly, induction of Kras^G12D^ mutation in acinar cells in mice leads to their transformation to PanIN lesions even in the absence of pancreas injury (Habbe et al., 2008).

Expression of mutant Kras in pancreatic acinar cells expedites their transformation to a duct-like phenotype by inducing local inflammation. Specifically, KrasG12D induces the expression of intercellular adhesion molecule-1 (ICAM-1), which serves as chemo-attractant for macrophages.

Infiltrating macrophages amplify the formation of KrasG12D-caused abnormal pancreatic structures by re-modeling the extracellular matrix and providing cytokines such as TNF-α. Depletion of macrophages or treatment with a neutralizing antibody for ICAM-1 in mice expressing oncogenic Kras under an acinar cell-specific promoter both resulted in a decreased formation of abnormal structures and decreased progression of acinar to ductal metaplasia to PanIN lesions (Liou et al., 2014). Of note, we have shown before that ICAM-1 is also expressed in acinar cells during pancreatitis (Zaninovic et al., 2000).

Both M1 and M2 macrophage phenotypes were found in the pancreatic tumor microenvironment. M1 macrophages are characterized by high expression of inducible nitric oxide synthase (iNOS), IL-10, major histocompatibility complex II (MHC-II), and low IL-12 among others. M2 macrophages express low level or no iNOS, IL-10, and MHC-II, and high levels of Arginase1, CD206, and C-C chemokine receptor 3 (CCR3). Greater levels of tumor-infiltrating M2 macrophages are significantly associated with shorter survival, whereas M1^high^/M2^low^ correlated significantly with longer survival and suggested using the M1/M2 ratio as independent prognosticator (Ino et al., 2013). Analysis of pancreatic cancer tissues from 36 patients showed that the number of infiltrating macrophages in tumor tissue was significantly greater in patients with metastases to lymph nodes compared to tumor tissue without metastasis (Gardian et al., 2012).

Co-culture with macrophages or with tumor associated macrophage-conditioned media significantly reduced apoptosis and activation of the caspase-3 pathway during gemcitabine treatment of pancreatic cancer cells pointing to a survival effect that the macrophages provide to cancer cells (Weizman et al., 2014). Also, reducing macrophage recruitment and activation in PDAC models in mice demonstrated improved response to gemcitabine compared with controls (Weizman et al., 2014). The data by Weizman et al. showed that decreasing macrophage recruitment augmented the response of PDAC to chemotherapy and that tumor associated macrophages induced up-regulation of cytidine deaminase (CDA), the enzyme that metabolizes gemcitabine following its transport into the cell (Weizman et al., 2014).

Liu et al. showed recently that co-culture with M2-polarized tumor associated macrophages up-regulated the expression of mesenchymal markers Vimentin and Snail and decreased the epithelial marker E-cadherin in pancreatic cancer cells (Liu et al., 2013). Furthermore, co-culture of tumor associated macrophages with pancreatic cancer cells increased proliferation and migration of the cancer cells. They demonstrated that TLR4 and IL-10 mediate the cross talk between tumor associated macrophages and cancer cells (Liu et al., 2013).

All these data indicate that a bidirectional interaction between cancer cells and macrophages determines the fate of their phenotypes, more or less metastatic and resistant to treatment for cancer cells, and pro-inflammatory or pro-cancer for macrophages.

## Interaction between macrophages and stellate cells

When quiescent pancreatic stellate cells are co-cultured with macrophage cell lines, the stellate cells showed morphological changes consistent with myofibroblast morphology (Shi et al., 2014). In the presence of Heparin-binding EGF (HB-EGF) many of the stellate cells co-cultured with macrophages were positive for their activation marker αSMA (Shi et al., 2014).

In the same study they found that PSCs have the potential to regulate macrophage function, inducing the production of multiple cytokines, partially through PSC production of IL-6 (Shi et al., 2014). However, caution should be taken as these studies were performed in cell lines and not in cells freshly isolated from animals or humans (Shi et al., 2014).

In a different study, culture of peripheral blood mononuclear cells (PBMC) with PSCs supernatants or IL-6 promoted PBMC differentiation into Myeloid-derived suppressor cells MDSC (CD11b^+^CD33^+^) phenotype and a subpopulation of polymorphonuclear CD11b^+^CD33^+^CD15^+^ cells (Mace et al., 2013). IL-6 was an important mediator as its neutralization inhibited PSC supernatant-mediated STAT3 phosphorylation and MDSC differentiation (Mace et al., 2013). Importantly, the level of cytokines produced by different PSCs was different suggesting different phenotypes of the PSCs and suggesting using these cytokines as markers for different sub-populations of PSCs.

**Figure 1:**
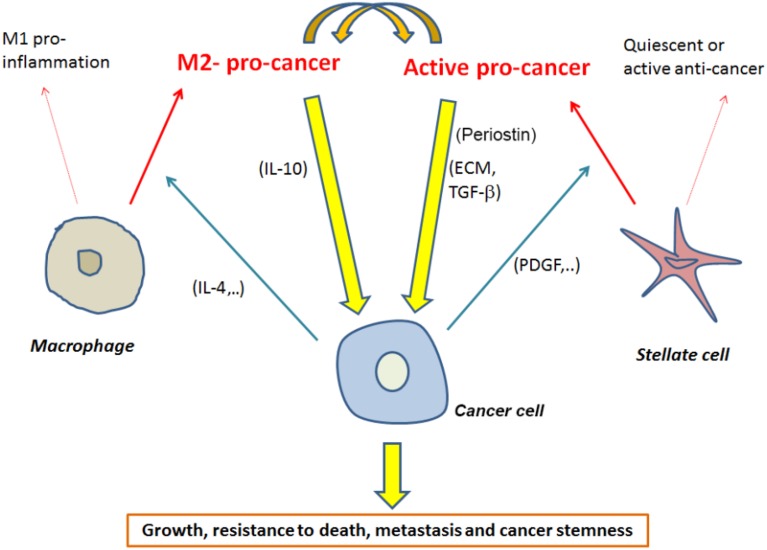
**Interactions between macrophages and stellate cells mediating promotion of pancreatic cancer with a short list of possible mediators of these interactions**.

Accumulating evidence indicate that targeting PSCs and/or macrophages needs to be focused on targeting specific sub-populations of these cells. Even though M2 macrophage populations are a good target, further studies are needed to determine markers of the pro-cancer PSCs (Figure 1).

Multiple studies have tried targeting pathways rather than targeting the whole cell populations. Therapies against molecules or pathways such as vascular endothelial growth factor (VEGF) (Taeger et al., 2011), sonic hedgehog (Olive et al., 2009), and hyaluronic acid (Provenzano et al., 2012) are now tested. Other studies including our work suggest that the IL-6 and STAT3 pathway is a relevant target due to its constitutive activation in the pancreatic cancer cells, immunosuppressive cells, and in PSC within the stroma (Corcoran et al., 2011; Lesina et al., 2011; Huang et al., 2012).

In summary, we have gained a lot of knowledge about the interactions between cancer cells and cells present in the tumor microenvironment, especially the interactions between cancer cells, macrophages, and PSCs. Ample work is needed to understand how cells in the tumor microenvironment regulate PSCs phenotype. Periostin could be is a good candidate to differentiate between different populations of PSCs.

### Conflict of interest statement

The authors declare that the research was conducted in the absence of any commercial or financial relationships that could be construed as a potential conflict of interest.
